# The Roles of Sea-Ice, Light and Sedimentation in Structuring Shallow Antarctic Benthic Communities

**DOI:** 10.1371/journal.pone.0168391

**Published:** 2017-01-11

**Authors:** Graeme F. Clark, Jonathan S. Stark, Anne S. Palmer, Martin J. Riddle, Emma L. Johnston

**Affiliations:** 1 Evolution and Ecology Research Centre, School of Biological, Earth and Environmental Science, University of New South Wales, Sydney, New South Wales, Australia; 2 Australian Antarctic Division, Department of Environment, Kingston, Tasmania, Australia; Auckland University of Technology, NEW ZEALAND

## Abstract

On polar coasts, seasonal sea-ice duration strongly influences shallow marine environments by affecting environmental conditions, such as light, sedimentation, and physical disturbance. Sea-ice dynamics are changing in response to climate, but there is limited understanding of how this might affect shallow marine environments and benthos. Here we present a unique set of physical and biological data from a single region of Antarctic coast, and use it to gain insights into factors shaping polar benthic communities. At sites encompassing a gradient of sea-ice duration, we measured temporal and spatial variation in light and sedimentation and hard-substrate communities at different depths and substrate orientations. Biological trends were highly correlated with sea-ice duration, and appear to be driven by opposing gradients in light and sedimentation. As sea-ice duration decreased, there was increased light and reduced sedimentation, and concurrent shifts in community structure from invertebrate to algal dominance. Trends were strongest on shallower, horizontal surfaces, which are most exposed to light and sedimentation. Depth and substrate orientation appear to mediate exposure of benthos to these factors, thereby tempering effects of sea-ice and increasing biological heterogeneity. However, while light and sedimentation both varied spatially with sea-ice, their dynamics differed temporally. Light was sensitive to the site-specific date of sea-ice breakout, whereas sedimentation fluctuated at a regional scale coincident with the summer phytoplankton bloom. Sea-ice duration is clearly the overarching force structuring these shallow Antarctic benthic communities, but direct effects are imposed via light and sedimentation, and mediated by habitat characteristics.

## Introduction

The annual timing of sea-ice formation and departure is critical to the functioning of polar ecosystems, but in recent decades sea-ice dynamics have been changing. Higher temperatures and stronger winds have reduced the annual duration of Arctic sea-ice [1, 2], and within the next 30 years the Arctic Ocean is predicted to experience its first ice-free summer in recorded history [3]. Sea-ice in the West Antarctic Peninsula has been departing earlier and forming later each year for the past few decades [4–6], while temporal trends in continental Antarctica are spatially variable [7]. To predict consequences of changing sea-ice on polar biodiversity and ecosystem function we must establish relationships between sea-ice dynamics and biota, and recent studies have made progress to this end [8–10]. However, we now need to fine-tune the mechanistic basis of these relationships, and to identify which factors mediate their strength.

Sea-ice dynamics directly impact biota living on or in the ice, such as mammals [11] and microorganisms [12], but also affect subtidal ecosystems. Environmental conditions in benthic and pelagic marine ecosystems are highly dependent on sea-ice dynamics, since the annual period of sea-ice cover determines average subtidal conditions. Recent studies report strong response of benthic communities to change in sea-ice cover [10, 13, 14], although surveys are generally at a coarse taxonomic scale and there is little information on benthos/sea-ice associations for small or cryptic taxa. Given the high diversity and productivity of polar benthic communities [5], better understanding of their relationship to sea-ice is critical for predicting future polar biodiversity.

Of the ways in which sea-ice affects benthic communities, the most obvious is reduced solar irradiance [8, 15]. Decreased photosynthetically active radiation (PAR) inhibits photosynthesis in algae [16] and diatoms [17], and protects organisms from potentially harmful ultraviolet wavelengths [18, 19]. Light is pivotal in mediating competitive interactions between algae and invertebrates, and in shallow waters can dictate overall community structure [20]. The amount of light an area of seabed receives annually depends on the timing of sea-ice breakout relative to the annual solar cycle, since sunlight is strongly seasonal at high latitudes [8]. Sea-ice determines both the type and quantity of primary productivity fuelling shallow benthic ecosystems. Algae growing on the underside of sea-ice provide the benthos with small amounts of food [21], supplemented by particles advected from ice-free areas [22]. When sea-ice breaks out, however, pelagic phytoplankton bloom and dramatically increase total primary production [23, 24].

Sea-ice also mediates physical disturbance to the benthos by influencing both sedimentation and ice-scour. Sea-ice forms a barrier between the water column and the atmosphere, preventing wind-induced turbulence and water-column turnover to create relatively still, depositional environments, where flow is generally limited to tidal currents [25, 26]. In such conditions, particulate matter settles out of the water column and sediments accumulate on the seafloor, potentially imposing sedimentation stress on benthic taxa. Sediment may be from glacial or terrestrial sources, resuspended marine sediments, or organic matter produced in the water column. A major source of organic matter is the summer phytoplankton bloom, which typically occurs immediately after sea-ice breakout in response to the sudden increase in light [23]. The role of sea-ice in phytoplankton deposition is two-fold: the timing of sea-ice breakout determines when the phytoplankton bloom happens, and sea-ice cover then affects the rate of deposition.

Sea-ice attached to coast (‘fast-ice’) also prevents drifting ice-bergs from scouring the seabed and inflicting catastrophic physical disturbance [27]. Studies in West Antarctica have found that the frequency of ice-scour to be directly related to seasonal sea-ice duration [9], and that this process strongly influences the composition and structure of benthic communities [28]. Frequently scoured seabeds are in perpetual states of early colonisation [5], and late successional communities only occur at depth or in areas protected by continuous ice cover. Depth of seabed can affect the rate of ice-scour, since the draught of an iceberg may be too shallow to reach the seabed at a particular location, or too deep to avoid being grounded before it reaches that location. For seabed of a given depth, susceptibility to ice-scour is therefore related to the size distribution of ice-bergs in the local area.

Evidence of links between sea-ice duration and shallow benthic community structure is growing, although patterns vary between study regions. A long-term study from the Arctic showed that macroalgae increased in abundance as sea-ice duration shortened [10, 29]–a process also described in Clark et al. (2013). Two study areas in the West Antarctic Peninsula have experienced reduced sea-ice duration over the past decades, but different processes of ecological change have been identified at each location. At Potter Cove (King George Island, 62° S) retraction of sea-ice over 25-years led to macroalgal colonization [13], while at Rothera Station (Adelaide Island, 68° S) reduced sea-ice increased the frequency of iceberg-scour and resulted in more disturbed communities [5, 9]. In addition to variation in effects of sea-ice between locations, there is also likely to be within-site variation that is not yet understood. Small-scale local factors, such as habitat orientation and depth, might ameliorate or augment the effects of sea-ice on benthic communities, and there is a need to understand how habitat features mediate sea-ice effects.

Light, sedimentation, and disturbance to the benthos are likely to be mediated by habitat orientation and depth. Both light and sedimentation are vertically oriented processes, so their effects on benthic organisms should depend on the orientation of habitat relative to overhead inputs [30, 31]. Effects of sea-ice might also change with depth due to light attenuation [32]. PAR decreases at an exponential rate through the water column [33], so effects of sea-ice on primary production and UV exposure should also decrease with depth. The frequency of ice-scour is highest in the shallows and decreases with depth, since fewer icebergs are large enough to reach deeper seabed [9, 28]. Knowledge of the interactions between sea-ice and habitat characteristics in affecting benthos is important to improve predictions about how benthos may be affected by changing climate. In this paper we present a rare multidisciplinary dataset, combining environmental and biological data collected from a seasonally ice-covered region of Antarctic coast. We report a detailed survey of biota on subtidal boulders at 11 sites across a gradient of sea-ice duration, and consider biological trends in the context of local light and sedimentation.

## Materials and Methods

### Study area

**Fig 1:**
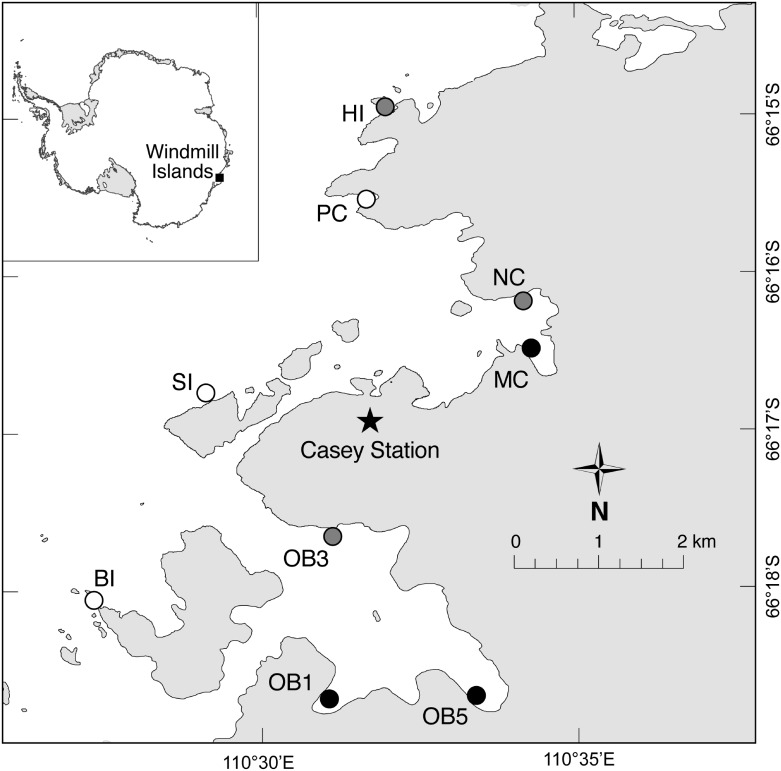
Map of study region in the Windmill Islands, located on the coast of Wilkes Land, East Antarctica. Casey Station is marked with a star. Abbreviated site names correspond to those in Table 1.

This study was conducted around Casey Station (66° 17’ S, 110° 32’ E), on the coast of Wilkes Land, East Antarctica (Fig 1). The subtidal landscape consists of a mosaic of granitic gneiss bedrock, boulder fields, and sediments [34]. Ambient sea temperature is approximately -1.8°C throughout the year. Access to these sites was permitted by the Australian Antarctic Division, and the work did not involve endangered or protected species.

### Light meters

Light meters were deployed on the seabed at eight sites around Casey Station (Fig 1, Table 1) between 1998 and 2004. These were Dataflow^™^ cosine corrected photosynthetic irradiance sensors (400–700 nm) [35], with data logged to Dataflow^™^ 292 data loggers or Odyssey^™^ integrated data loggers. Light meters were mounted upright on stainless-steel tripods or star pickets, and were positioned and secured on the seabed by divers at depths of 7 to 10 m. They were calibrated against three Li-Cor LI-190SA sensors (Lincoln, Nebraska, USA) for 1 w prior to deployment, and set to record ambient light levels every 20 min. Due to the deterioration in performance of light meters through time as a result of physical disturbance, shading or fouling, only the first 12 months of light data are presented. Raw data from light meters was converted into mol photons PAR m^-2^ yr^-1^.

**Table 1:** Summary of sea-ice conditions and types of data collected at each site (denoted by *x*). Site codes are used in Fig 1. Ice-rank is an ordinal number given to sites where biota were sampled, based on light measurements and long-term visual observations (NA refers sites where biota were not sampled). Lower numbers indicate later sea-ice breakout. Ice-zone is a classification is given to all sites, for categorical analyses and to describe unranked sites.

Site	Code	Ice-rank	Ice-zone	Biota	Light	Sedimentation
Brown Bay	BB	NA	Closed			*x*
Sparkes Bay 1	SP1	NA	Closed			*x*
Sparkes Bay 2	SP2	NA	Closed			*x*
O’Brien Bay 1	OB1	1	Closed	*x*	*x*	*x*
O’Brien Bay 5	OB5	2	Closed	*x*	*x*	*x*
McGrady Cove	MC	3	Closed	*x*	*x*	*x*
O’Brien Bay 2	OB2	4	Intermediate	*x*	*x*	
Newcomb Corner	NC	5	Intermediate	*x*	*x*	
Shannon Bay	SB	6	Intermediate	*x*	*x*	
O’Brien Bay 3	OB3	7	Intermediate	*x*	*x*	
Honkala Island	HI	8	Intermediate	*x*		
Beall Island	BI	9	Open	*x*		
Powell Cove	PC	10	Open	*x*		
Shirley Island	SI	11	Open	*x*	*x*	

### Sedimentation

Sediment flux was quantified by deploying sediment traps at six sites (Fig 1, Table 1 and S1 Table) for a range of intervals between December 2002 and December 2006. A total of 211 traps were deployed: 187 for periods of 9–89 days, 20 for periods of 260–375 days, and 4 for periods of 640–724 days. At each site, traps were deployed in inner and outer zones, where inner zones where closer to the shore and generally experienced later annual sea-ice breakout. Prior to deployment, traps were scrubbed with water and detergent, rinsed with Milli-Q water and placed in a 10% nitric acid bath for 24 hours, rinsed again with Milli-Q and sealed ready for deployment. Traps had an internal diameter of 5.4 cm and an internal height of 13.7 cm. No preservatives were used to keep the settling particles. Collected sediment material was transported to the laboratory at Casey Station and stored at 4°C until processed (within 24 hours of collection). Sediment material was twice filtered through pre-weighed 0.8 micron glass fiber filters and 0.45 micron cellulose acetate filters. Filters were then placed on individual petri dishes, oven dried at 60°C, cooled in a desiccator and their dry weights recorded.

### Biological survey

Divers collected boulders from 11 sites across a gradient of seasonal sea-ice cover (Fig 1, S2 Table). At each site divers collected eight boulders (mean surface area per boulder ± SE = 344 ± 19 cm^2^) from two depths, 6 and 12 m, 100–200 m apart. There was no significant difference in the size of boulders collected from different sites or depths (2-factor ANOVA, *P* > 0.05). Surveys were conducted across two austral summers (January–February 2006 and October–December 2006; Table 1) but given the large temporal scales over which these communities develop [36] we did not expect strong differences between summers. To test this we sampled one site (O’Brien Bay 1) twice, once in each summer, and did not detect significant differences in community structure (ANOSIM, R = 0.029, *P* = 0.32).

Boulders were kept in aerated local seawater at -2°C, and communities were censused live under a dissecting microscope. Quadrats (2 x 2 cm) were randomly placed on a boulder, and all taxa occurring within a quadrat were recorded. On each boulder we sampled six quadrats on horizontal (up-facing) surfaces and six on vertical (side-facing) surfaces, giving a total of 48 cm^2^. Down-facing surfaces were not included because this surface was bare on boulders at silty sites. The placement of quadrats was constrained to be > 1 cm from any edge and at least 2 cm from another quadrat. Counts were made of serpulid and spirorbid polychaetes, and percent cover was estimated for all other taxa. All cheilostomate bryozoans were identified to species. Other taxa were categorised into morphospecies. Voucher specimens were collected and preserved. Sponges and calcified bryozoans were preserved in 80% ethanol with Milli-Q water, and other taxa were preserved in 7% formalin in seawater.

### Statistical analyses

**Fig 2:**
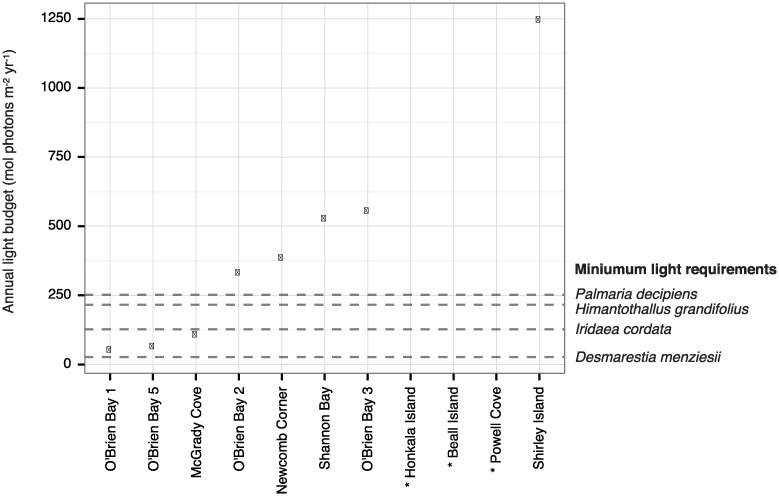
Annual light budgets (ALB) at the seabed. Points represent ALB recorded by light meters during the first year of deployment, and dashed lines are estimated minimum annual light requirements of four species of local algae (from Clark et al. 2013). Algae species are expected to be viable at sites where the ALB is above their minimum requirement. Light measurements are not available for three sites marked with *, but from long-term visual observation we know their annual timing of sea-ice departure and therefore approximate light budgets.

Sites were ranked according to their sea-ice duration (‘Ice-rank’, Table 1), as determined by annual light budgets (Fig 2) and visual observations by Riddle and Stark since 1997. For univariate analysis, ice-rank was used as a continuous variable to represent the gradient in sea-ice cover, because light measurements (and therefore date of sea-ice breakout) were not available for three of the eleven sites. For multivariate analysis (PERMANOVA), sites were classified into one of three ice-cover zones (‘Ice-zone’, Table 1), since this analysis requires categorical predictors. Categories were open, intermediate, and closed, where closed sites are generally ice-covered >11 mo yr^-1^, intermediate sites 7–11 mo yr^-1^, and open sites <7 mo yr^-1^.

For boulder communities, multivariate community-level effects of sea-ice cover (open, intermediate and closed), depth (6 and 12 m) and substrate orientation (horizontal and vertical) were analysed with PERMANOVA [37] using PRIMER v.6 [38]. The design included the above fixed factors, and two nested random effects of site (nested within sea-ice cover) and boulder (nested within site). Data were fourth-root transformed to reduce the influence of abundant taxa, and a resemblance matrix constructed using Bray-Curtis similarity. *P*-values were obtained with 999 permutations.

Effects of sea-ice cover (ice-rank), depth and substrate orientation on the abundance and diversity of individual taxa on boulders were analysed with generalized linear mixed models (GLMM) [39]. Spatial autocorrelation between surfaces within boulders, and boulders within sites, was modelled with random intercepts [40]. Percentage cover was modelled as a binomial response, and species richness and density were modelled as Poisson responses.

Sediment trap data collected in summer were analysed with a GLMM, testing the relationship between deployment duration (number of days), position in a bay (inner or outer), and sediment flux (g m^-2^ d^-1^). The response was modeled with a Gaussian distribution with log link function, and Site (bay) was modeled as a random intercept to account for autocorrelation within sites. Only data from traps deployed during summer were statistically analysed.

All GLMMs were conducted using the ‘lme4’ package v.1.1–7 [41] in R v.3.1.1 (R Development Team 2014). Parameters were estimated with Laplace approximations [42], and *P*-values obtained with *Z*-tests. Plots of residuals were inspected for homoscedasticity.

## Results

### Light at the seabed

Annual light budgets on the seabed ranged from 54 to 1248 mol photons m^-2^ yr^-1^ (Fig 2), and were positively related to the duration of the annual ice-free period. Based on published minimum annual light budgets for four species of local algae (Clark et al. 2013), algae should be capable of surviving at eight of the eleven sites where we sampled biota (Fig 2). Light measurements are not available for three sites, but from visual observations since 1997 we know their annual timing of sea-ice departure, and therefore we assume that their light budgets were similar to those from Shirley Island.

### Sedimentation

**Fig 3:**
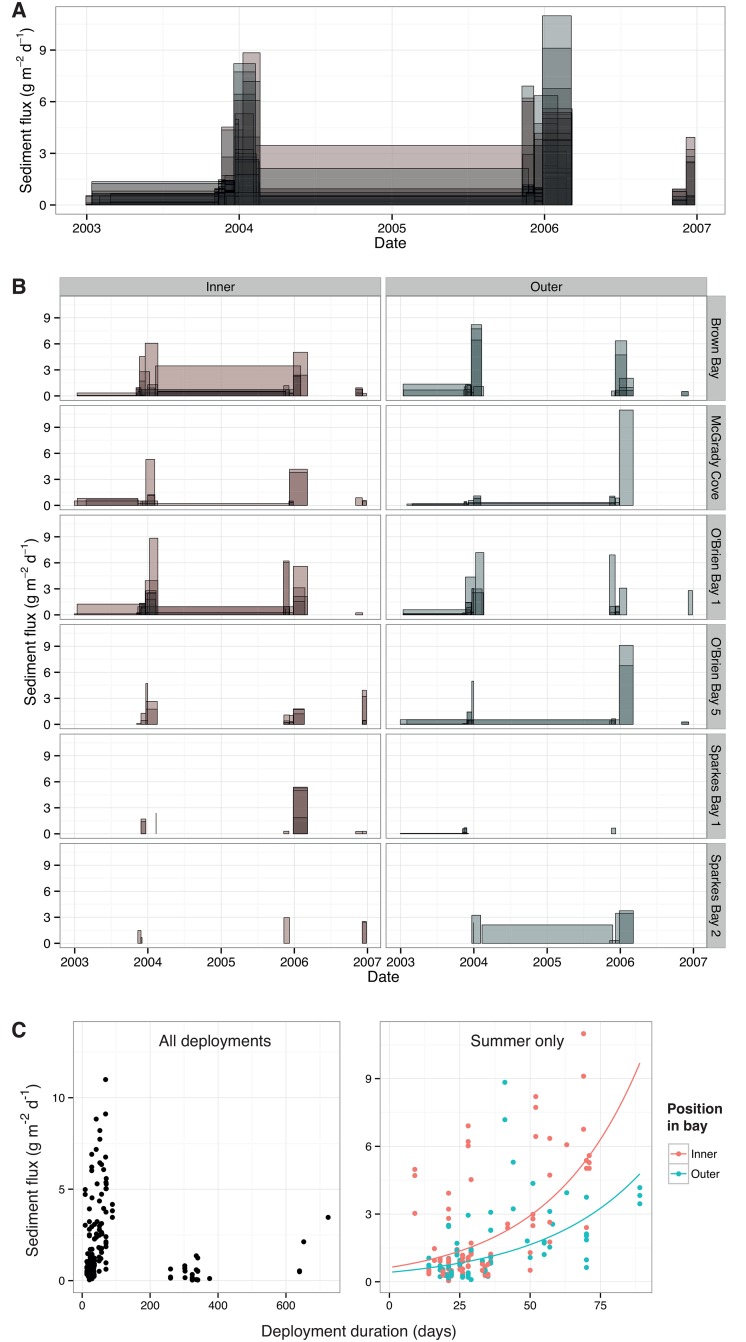
Sedimentation flux through time. (A) Polygons are individual sediment traps, with height showing average daily sediment flux and width showing deployment duration. Colour represents position in bay: red = inner, green = outer. (B) As above, but data are partitioned into sites and position within bay. (C) Plots of average daily sediment flux vs. deployment duration. Left-hand plot shows all traps, and right-hand plot shows traps deployed during summer only. In right-hand plot, fitted lines are predicted values of GLMM for each position in bay.

Sediment trap data show peaks in deposition around mid-summer (Fig 3A), which did not appear to be related to site-specific sea-ice dynamics (Fig 3B). Sites where sediment flux was measured are typically ice-covered until late summer, 1–2 months after peak sediment deposition was observed. However, sedimentation rates were higher in the inner area of bays, relative to outer area (Table 2, Fig 3C), suggesting a spatial relationship between sediment flux and sea-ice cover. For summer deployments, there was a positive relationship between the length of trap deployment duration and average daily sediment flux (Table 2, Fig 3C). Supplementary Table 1 shows minimum, mean, and maximum, sediment fluxes for each site and position in bay.

**Table 2:** Estimates, standard errors (SE), *t*-values and *P*-values for fixed effects in GLMM testing the effects of deployment duration (number of days) and position (inner or outer area of a bay) on daily sediment flux (g m^-2^ d^-2^). Random intercepts were allowed per site (bay). Estimates are in log units. Significant differences (P < 0.05) are denoted in bold.

	Estimate	SE	*t*	*P*
(Intercept)	-0.460	0.009	-52.560	**<0.001**
Duration	0.031	0.003	11.380	**<0.001**
Position	-0.411	0.009	-47.680	**<0.001**
Duration: Position	-0.003	0.002	-1.400	0.163

### Boulder communities

**Fig 4:**
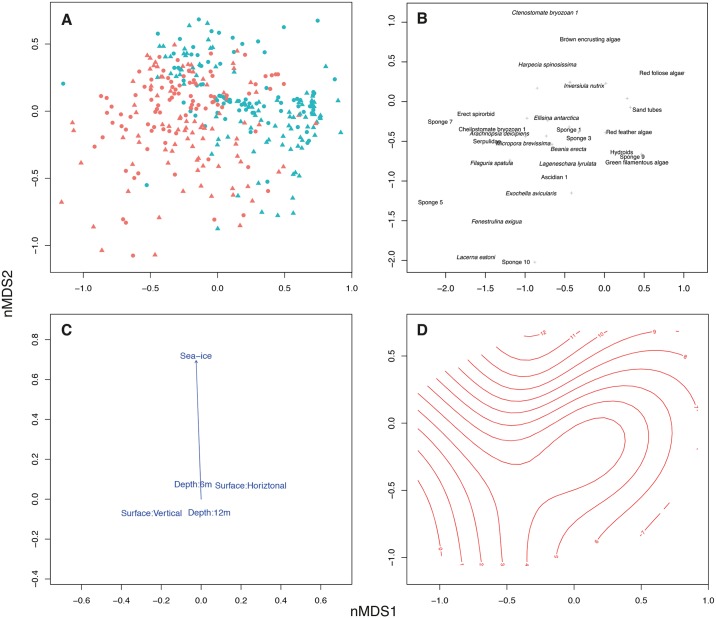
Multivariate patterns in boulder communities. (A) Multidimensional scaling plot (nMDS) based on Bray-Curtis similarity, showing boulder communities as points. Shapes represent depths (circles = 6 m, triangles = 12 m), colours represent orientations (red = Vertical, green = Horizontal). Axes are scaled such that 1 unit equates to a doubling in dissimilarity between samples. (B) Correlations between species and nMDS axes. Where species positions overlap, the more abundant species is displayed by name and other species appear as grey crosses. (C) Vector correlation between continuous environmental covariate (sea-ice rank) and nMDS axes, and position of centroids of factor levels. (D) Contour plot of sea-ice rank, fitted by GAM to nMDS axes.

In total we recorded 42 taxa, including algae, polychaetes, bryozoans (mostly cheilostomate), hydroids, sponges and ascidians (S3 Table). Multivariate analyses found that communities differed between ice-cover zones, but differences depended on substrate orientation (Table 3, Fig 4). Vertical substrates (lower-left quadrat of nMDS, Fig 4A) were characterized by a diversity of bryozoa and sponges, while horizontal substrates (upper-right quadrat of nMDS, Fig 4A) contained algae, sand tubes, and a different suite of bryozoa (Fig 4B). There was a strong gradient in community structure correlated with sea-ice duration (Fig 4C), but a surface plot shows that change with sea-ice is not linear in relation to nMDS axes (Fig 4D). Change is relatively linear in the lower-left quadrat of the nMDS, the region correlated with vertical substrates, but diverges in the upper-right quadrat, the region correlated with horizontal substrates (Fig 4D). This represents a strong gradient in community structure relative to sea-ice on horizontal surfaces, compared to comparatively steady change with sea-ice on vertical surfaces. Horizontal surfaces are correlated with sand tubes in areas of greater sea-ice duration, but brown encrusting algae in areas of shorter sea-ice duration. Community structure also varied with depth (Table 3), but in the same direction as sea-ice relative to nMDS axes (Fig 4C).

**Table 3:** Permutational multivariate analysis of variance (PERMANOVA) testing for differences in community structure with respect to ice cover, depth and orientation. Significant differences (*P* < 0.05) are denoted in bold. Data are fourth-root transformed, and distances were based on the Bray-Curtis similarity index.

Source	*df*	*MS*	*Pseudo-F*	*P*
*Between Sites(Ice)*				
Ice	2	53630	5.8866	**0.001**
Depth	1	10699	3.4144	**0.009**
Orientation	1	25307	12.795	**0.001**
Depth x Orientation	1	679.18	0.58571	0.762
Ice x Depth	2	4410.6	1.4075	0.197
Ice x Orientation	2	5159.3	2.6085	**0.015**
Ice x Depth x Orientation	2	888.7	0.7664	0.673
*Within Sites(Ice)*				
Site(Ice)	6	9110.5	10.71	**0.001**
Site(Ice) x Depth	6	3133.6	3.6838	**0.001**
Site(Ice) x Orientation	6	1977.8	3.4492	**0.001**
Site(Ice) x Depth x Orientation	6	1159.6	2.0222	**0.003**
*Boulder (blocking)*				
Boulder(Site(Ice) x Depth)	126	850.65	1.4835	**0.001**
Error	126	573.42		

**Fig 5:**
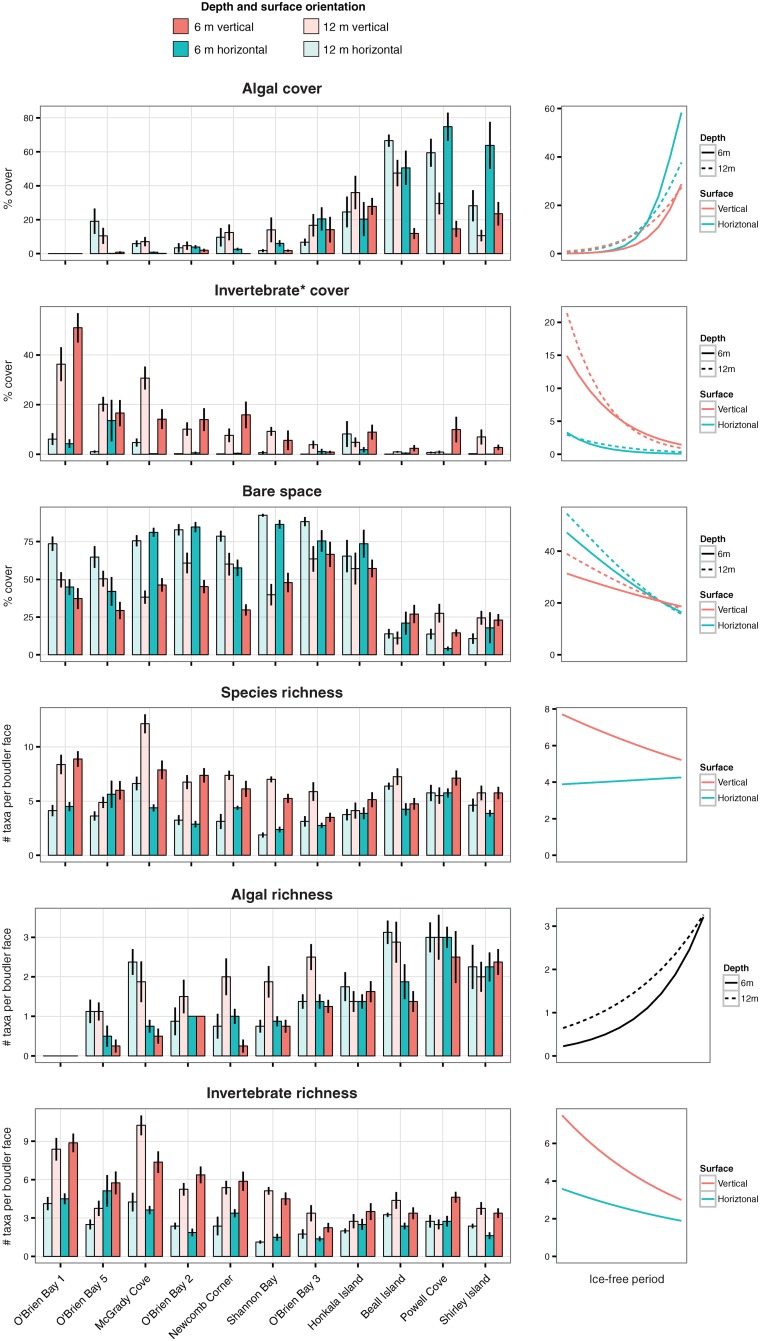
Percentage cover of algal, invertebrates, and bare space, and measures of diversity. Left-hand plots show data from all sites, depths and orientations. Bars show the mean value, and error bars show standard error. Right-hand plots show predicted values of significant effects detected by GLMM.

There were very strong trends in the total cover of algae and invertebrates relative to sea-ice duration, with the exception of two highly abundant invertebrates–*Inversiula nutrix* and spirorbid polychaetes. Algal cover was higher on horizontal surfaces within sites classified as having shorter sea-ice duration (Table 4, Fig 5), where the incidence of light is greatest. Invertebrate cover (excluding *I*. *nutrix* and spirorbids) showed the opposite pattern, particularly on vertical surfaces at 12 m depth (Table 4, Fig 5). There was less bare space in areas of shorter sea-ice duration, with an apparent step-change to low bare space at sites in the open zone (Table 4, Fig 5). Species richness was higher on vertical than horizontal substrates, although there were no significant trends in relation to sea-ice duration (Table 4, Fig 5). Invertebrate and algal richness did, however, increase and decrease with sea-ice duration, respectively (Table 4, Fig 5).

**Table 4:** Estimates, standard errors (SE), and *P*-values for fixed effects in GLMMs, testing the effects of sea-ice cover, depth, and substrate orientation on biota. Ice is a semi-quantitative continuous variable, derived by ranking sites in decreasing order of seasonal sea-ice cover. Random intercepts were allowed per Boulder nested in Site, and base levels for categorical fixed effects were Depth (6 m) and Surface (Horizontal).

	Algal cover			Invertebrate* cover			Bare space			Species richness ^δ^			Algal richness ^δ^			Invertebrate richness ^δ^		
	Estimate	SE	*P*	Estimate	SE	*P*	Estimate	SE	*P*	Estimate	SE	*P*	Estimate	SE	*P*	Estimate	SE	*P*
(Intercept)	-7.368	0.738	**<0.001**	-1.494	0.393	**<0.001**	-0.716	0.320	**0.025**	2.025	0.140	**<0.001**	-2.066	0.484	**<0.001**	2.112	0.148	**<0.001**
Ice	0.587	0.104	**<0.001**	-0.248	0.059	**<0.001**	-0.069	0.047	0.145	-0.038	0.021	0.069	0.293	0.063	**<0.001**	-0.091	0.023	**<0.001**
Depth	2.403	0.488	**<0.001**	0.531	0.374	**<0.001**	0.375	0.143	**0.009**	0.112	0.118	0.343	1.653	0.397	**<0.001**	-0.015	0.126	0.905
Orientation	-0.552	0.235	**0.019**	-1.512	0.136	**<0.001**	0.752	0.063	**<0.001**	-0.607	0.139	**<0.001**	0.539	0.444	0.224	-0.616	0.150	**<0.001**
Ice: Depth	-0.224	0.066	**0.001**	-0.091	0.058	0.118	-0.040	0.022	0.067	-0.002	0.018	0.921	-0.148	0.048	**0.002**	-0.001	0.021	0.977
Ice: Orientation	0.163	0.026	**<0.001**	-0.131	0.035	**<0.001**	-0.082	0.010	**<0.001**	0.033	0.021	0.116	-0.047	0.053	0.373	0.003	0.025	0.910
Depth: Orientation	-0.092	0.268	0.732	-0.803	0.200	**<0.001**	-0.048	0.085	0.572	-0.250	0.197	0.204	-0.956	0.541	0.077	-0.305	0.218	0.161
Ice: Depth: Orientation	-0.062	0.030	**0.042**	0.252	0.044	**<0.001**	0.005	0.014	0.729	0.030	0.029	0.299	0.084	0.066	0.201	0.050	0.036	0.162
	*Inversiula nutrix*			Spirorbidae ^δ^			Sand tubes			Sponges			Encrusting algae			Arborescent algae		
	Estimate	SE	*P*	Estimate	SE	*P*	Estimate	SE	*P*	Estimate	SE	*P*	Estimate	SE	*P*	Estimate	SE	*P*
(Intercept)	-1.494	0.571	**0.009**	0.480	0.748	0.521	-2.900	0.001	**<0.001**	-4.052	0.589	**<0.001**	-11.993	1.320	**<0.001**	-7.171	0.997	**<0.001**
Ice	<0.001	0.084	0.999	0.023	0.110	0.831	-0.421	0.001	**<0.001**	-0.113	0.089	0.205	0.928	0.175	**<0.001**	0.381	0.142	**0.007**
Depth	-1.576	0.270	**<0.001**	-0.784	0.282	**0.005**	0.297	0.001	**<0.001**	1.017	0.613	0.097	6.282	0.971	**<0.001**	-1.608	0.796	**0.043**
Orientation	0.188	0.076	**0.014**	-1.167	0.225	**<0.001**	1.184	0.001	**<0.001**	-1.366	0.332	**<0.001**	-0.487	0.455	0.285	0.514	0.323	0.112
Ice: Depth	0.130	0.039	**0.001**	0.056	0.038	0.142	0.068	0.001	**<0.001**	-0.182	0.098	0.062	-0.624	0.121	**<0.001**	0.257	0.103	**0.013**
Ice: Orientation	-0.174	0.012	**<0.001**	0.054	0.027	**0.045**	0.005	0.001	**<0.001**	-0.079	0.057	0.166	0.176	0.047	**<0.001**	-0.020	0.040	0.623
Depth: Orientation	-1.815	0.157	**<0.001**	-1.964	0.545	**<0.001**	0.103	0.001	**<0.001**	-0.315	0.440	0.475	0.104	0.475	0.827	-4.045	0.568	**<0.001**
Ice: Depth: Orientation	0.263	0.020	**<0.001**	0.239	0.056	**<0.001**	-0.080	0.001	**<0.001**	0.159	0.076	**0.036**	-0.081	0.050	0.104	0.408	0.067	**<0.001**

Invertebrate* excludes *Inversiula nutrix* and Spirorbidae.

^δ^ denotes Poisson GLMM and Estimates in log units; all other variables used binomial GLMM and Estimates are in logit units. Significant differences (*P* < 0.05) are denoted in bold.

**Fig 6:**
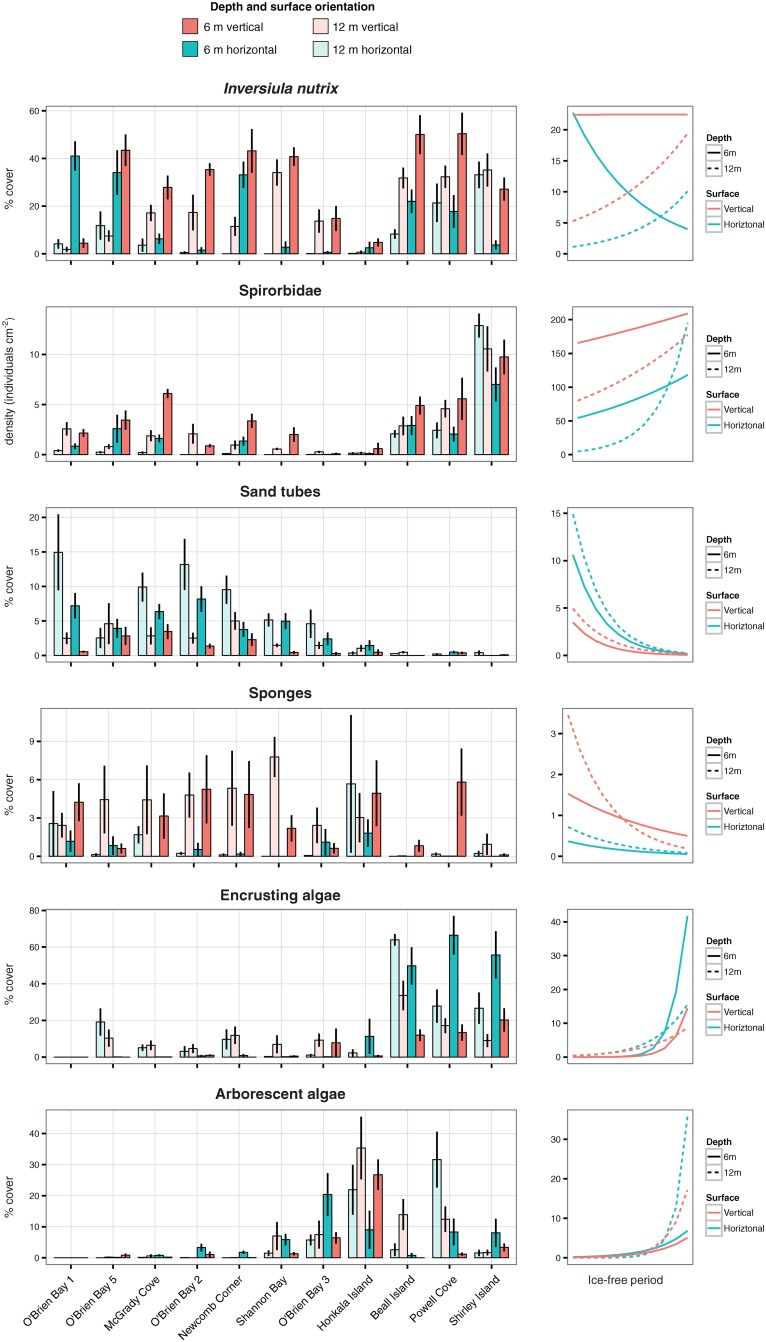
Percentage cover or density of dominant taxa and groups. Left-hand plots show data from all sites, depths and orientations. Bars show the mean value, and error bars show standard error. Right-hand plots show predicted values of significant effects detected by GLMM.

The bryozoan *Inversiula nutrix* was by far the most abundant taxon, and together with spirorbid polychaetes showed different trends than most other invertebrates. *I*. *nutrix* was abundant at almost all sites (Fig 6), although the type of habitat it dominated varied with sea-ice duration. In areas of extended sea-ice duration it dominated horizontal surfaces, but dominance shifted to vertical surfaces in areas with shorter sea-ice duration (Table 4, Fig 6). Spirorbid polychaetes (mostly *Spirorbis nordenskjoldi*) were the second most abundant invertebrate taxa, and were considerably more abundant at open sites than sites with more sea-ice cover. Most sand tubes were usually inhabited by tanaids of the genus *Nototanais*, but some housed other mobile invertebrates and terrebellid polychaetes. Sponge cover was highly variable between sites, but showed an overall trend of greater cover with longer sea-ice duration. Encrusting algae were most abundant in the open zone, whereas arborescent algae peaked in cover at sites with intermediate sea-ice duration (Table 4, Fig 6).

## Discussion

This unique synthesis of light, sedimentation, and biological data provides new insight into processes shaping nearshore Antarctic benthos. Data demonstrate that spatial variation in sea-ice duration creates opposing gradients of light and sedimentation, which in turn shape the structure and composition of shallow Antarctic benthic communities. Dominance shifted from algae at sites with shorter sea-ice duration, more light and less sedimentation, to sessile invertebrates at sites with longer sea-ice duration, less light and higher sedimentation. Importantly, however, these effects of sea-ice were mediated by habitat characteristics, such as substrate orientation and depth. The relationship between sea-ice duration and benthos was stronger on horizontal than vertical surfaces, which is explicable in that light and sedimentation are vertically oriented and affect horizontal more than vertical surfaces. The dependency of sea-ice effects on substrate orientation supports the model that light and sedimentation are two major pathways by which sea-ice duration shapes shallow coastal benthic communities. Shallow, horizontal habitats are most tightly linked with sea-ice dynamics, and their biota may be useful sentinels of climate change in nearshore polar ecosystems.

### Environmental gradients with sea-ice

Sites that are ice-free for ~ 10% of the year have annual light budgets more than 20 times lower than sites ice-free for ~ 50% of the year; a nonlinearity caused by the marked seasonality in solar irradiance at high latitudes [8]. Ice-free days receive far more light in summer than in winter, so the annual light budget is highly sensitive to the number of ice-free days around midsummer, and relatively insensitive to the number of ice-free days closer to winter [8]. Most variation in the timing of sea-ice breakout in the Windmill Islands occurs during summer months, so sites represent a pronounced gradient in annual light despite only differing in their date of sea-ice breakout by several weeks or months. Other factors that mediate effects of sea-ice on subtidal light are ice thickness [43], snow cover, dust, and coastal topography, though these are usually minor in comparison to sea-ice breakout.

Sedimentation was also related to sea-ice cover, but in a different way to light. Sediment flux was higher in the inner than outer areas of sites, and therefore spatially covaries with the duration of sea-ice cover. However, the timing of peak deposition was decoupled from the timing of sea-ice breakout. Most deposition occurred in late December, around midsummer, yet sites where sedimentation was measured typically retain their sea-ice until late January or February. This implies that most sedimentation originates from the summer phytoplankton bloom—an annual event in which suspended microalgae rapidly proliferate [44] then settle to the seabed. The advection of phytoplankton to under-ice areas [22] then creates a region-wide deposition event, whose timing is independent of site-specific sea-ice breakout. However, the rate of phytoplankton settling from the water column *is* likely related to sea-ice breakout, since turbulence and flow are much reduced under sea-ice [45]. Therefore, we suggest that suspended sediment load in the water column does not vary with sea-ice duration, but that deposition and retention do, and are positively related to sea-ice duration. The positive correlation between the sediment trap deployment period and average daily flux also implies that deposition occurs in pulse events not captured by some of the shorter deployments, which is consistent with a bloom event. Some sediment may also arrive via terrestrial runoff during the summer melt, although this is less likely when sites are covered by sea-ice, as they were when deposition peaked in our sampling.

Comparison of Windmill Island sediment fluxes with other published Antarctic near-coastal studies reveal that sediment fluxes in the region are very low. The mean summer flux for the Windmill Islands varies between 0.55 and 7.61 g m^-2^ day^-1^ in comparison with 43, 23.6 and 10 g m^-2^ day^-1^ at Johnson’s Dock, Livingston Island [46]; Marian Cove [47], King George Island and Potter Cove, King George Island [48], respectively with higher sediment fluxes reported at Potter Cove after 1992. One explanation is that these published studies are located on islands and in the region of the South Shetlands near the Antarctic Peninsula. The Antarctic Peninsula has a much milder and more maritime climate than continental Antarctica, whereas our study locations were adjacent to continental Antarctica and may therefore be influenced by different processes.

Research programs established in different regions of Antarctica have focused on different processes by which sea-ice affects benthos. Studies in Potter Cove (West Antarctic Peninsula) found that glacial melting allowed macroalgae to colonize newly ice-free areas, but also report reduced light penetration due to increased terrigenous sediment input from land and more frequent ice scour [13]. Other research programs on the West Antarctic Peninsula focus on the change in the frequency of iceberg scour with sea-ice cover, and consider this the driving force affecting benthic communities [5, 9, 49–51]. The structure of benthos in our study area suggests that ice-scour in the Windmill Islands is not as frequent as at Antarctic Peninsula study locations, possibly due to more glaciation and icebergs and a more maritime climate on the Peninsula (more exposure to waves, currents, turbulence) against an environment which has more stable ice, with cooler temperatures and the prevalence of offshore winds as in many areas of continental Antarctica. Given the slow recruitment and growth of Antarctic benthic fauna [36] our study sites did not appear to have been recently disturbed, and bare space (an indicator of disturbance history) was lowest at sites in the open zone, where exposure to ice-scour should be greatest. It appears that light and sedimentation are the dominant physical processes by which sea-ice affects benthos in the Windmill islands, but in the region of the Antarctic Peninsula these may be secondary to iceberg disturbance.

### Biological gradients with sea-ice

There was a strong biological gradient transitioning from invertebrate to algal dominance in the direction of decreasing sea-ice duration, but the nature of change depended on substrate orientation and depth. Horizontal surfaces were dominated by algae in areas of shorter sea-ice duration, and sand tubes and the bryozoan *Inversiula nutrix* in areas of longer sea-ice duration. Vertical surfaces were dominated by invertebrates regardless of sea-ice, but the identity of invertebrates changed along the sea-ice gradient. Communities on horizontal substrate showed stronger correlations to sea-ice duration than those on vertical substrate, consistent with expected response to greater exposure to light and sedimentation. In temperate and tropical systems, non-coral invertebrates tend to occupy shaded, vertical, or down-facing habitats [20, 31, 52], since they can be negatively affected by ultraviolet light [18] or outcompeted by algae in well-lit areas [53, 54]. Effects of sedimentation also vary with orientation [55], as sediment is more likely to settle and accumulate on horizontal than vertical substrates.

The relative effects of light and sedimentation can be inferred from changes in biota in relation to orientation, in areas of longer vs. shorter sea-ice duration. Assuming these physical factors affect horizontal more than vertical substrates, differences in biota between orientations at open sites (high light, low sedimentation) most likely reflect changes in light, while differences at closed sites (low light, high sedimentation) are more likely related to sedimentation. With this rationale, light in our study area drives a gradient between algae and invertebrates, whereas sedimentation facilitates fauna with sand tubes, and differentiates taxa based on morphology or other mechanisms of sediment tolerance. Light and sedimentation may also have interactive effects since negative consequences of reduced light on algae can be exacerbated under heavy sedimentation [56], and such an interaction may have contributed towards the sharp decline in algae with increased sea-ice duration.

Habitat use by the most abundant species, *Inversiula nutrix*, appears related to both light and sedimentation. It is the only sessile invertebrate taxa able to reach substantial cover on horizontal substrate at closed sites, implying a degree of tolerance to sedimentation. In contrast, at open sites it dominates vertical substrate, and is rare on horizontal substrate. This switch in habitat use from horizontal to vertical substrate as sea-ice duration shortens is likely driven by tolerance to sedimentation giving way to out-competition by algae. It may also be more tolerant of UV light than other invertebrates, since few of the invertebrate taxa found in the closed ice-cover zone were present in the open ice-cover zone. Spirorbid polychaetes (mostly *Spirorbis nordenskjoldi*) were the other invertebrate taxa to show contrasting distributions to the majority of other invertebrates, being most abundant in the open zone. Interestingly, both of these taxa were by far the most abundant in the study region, and their ecological success may be related to an ability to exploit niches unavailable to other invertebrates.

Overall trends in algal cover are clearly driven by light, but sedimentation may contribute to more subtle differences in the distribution of algal functional groups. Both encrusting and arborescent algae were negatively correlated with sea-ice duration, though cover of arborescent algae peaked in areas with some sea-ice cover. This could reflect greater tolerance to sedimentation in arborescent vs. encrusting morphologies, since arborescent foliage are raised off the substratum and remain clear of sediment accumulation [56]. Arborescent algae might experience reduced competition with encrusting algae by occupying areas of higher sedimentation. Similar patterns have been found in temperate systems, where articulated coralline algae are advantaged relative to encrusting coralline under higher rates of sedimentation [57]. Alternatively, encrusting algae may be more tolerant of the canopy-forming macroalgae that occur in areas of shorter sea-ice duration. Macroalgae impose a range of effects on the understorey environment, including changes in light [57], flow [58], sedimentation [56], and physical abrasion by canopy foliage [59]. Physical abrasion favours encrusting over arborescent forms in understorey communities in temperate Australia [59], and similar processes may be happening in Antarctica.

There was an intriguing peak in bare space in the intermediate ice-cover zone, which may reflect disturbance conditions or poor habitat quality. One hypothesis to explain the pattern is that iceberg scour in the intermediate ice-cover zone causes regular invertebrates mortality, but in contrast the open ice-cover zone, light is insufficient to support algal growth. Such conditions would create habitat unfavourable to both invertebrates and algae. The spatial mosaic of algae in the open zone suggests that iceberg scour is occurring and has ecological consequences [60], but rates of ice-scour have not been quantified in this region like has been done in other parts of Antarctica [29]. Quantifying ice-sour, or conducting a reciprocal transplants of boulder communities between sea-ice cover zones, would aid in understanding the mechanism driving this pattern.

In summary, we found strong links between sea-ice duration and benthic community structure, and the nature of change was consistent with variation in light and sedimentation. Both light and sedimentation vary with sea-ice duration, and their effects on biota are mediated by the depth and orientation of habitat. Interactions between overhead sea-ice dynamics and benthic habitats undoubtedly contribute to the diversity of shallow Antarctic benthic communities, and understanding these interactions is critical to forecasting climate-related change. If climate change lengthens the period for which some areas are ice-free per year, species distributions are likely to shift accordingly. Taxa currently restricted to areas of prolonged ice cover are at risk of local extinction, which is ecologically significant because these areas contained a suite of invertebrates not found in areas of shorter sea-ice duration. Such change in benthic communities in response to sea-ice duration has recently been observed in both the Arctic [10] and Antarctic [13, 61], and are predicted to become more frequent with continuing change in sea-ice dynamics [8]. Moreover, since much of the Antarctic coast lies in a relatively narrow latitudinal band, large areas of shallow habitat may be vulnerable within an ecologically-brief period of time [62]. Communities on horizontal habitats are likely to be useful indicators of climate change in shallow polar habitats, since these bear the strongest correlations with sea-ice duration.

## Supporting Information

S1 Table Sampling details of sediment traps, and minimum, mean and maximum sediment flux for each site during the study period. The number of deployments represents the number of sediment traps deployed at one site, with 2–5 sediment traps deployed at any one time. Traps were deployed for various time intervals between 08/12/2002 and 30/12/2006.
(DOCX)

S2 Table Details of sampling sites and times for the surveys of biota. At each site we collected 16 boulders, 8 from 6 m depth and 8 from 12 m.
(DOCX)

S3 Table Taxa recorded in boulder survey. (DOCX)
