# Correction: The Roles of Sea-Ice, Light and Sedimentation in Structuring Shallow Antarctic Benthic Communities

**DOI:** 10.1371/journal.pone.0173939

**Published:** 2017-03-09

**Authors:** The PLOS ONE Staff

In Fig 2, the data points do not display properly. Please see the correct Fig 2 here. The publisher apologizes for the error.

**Fig 2:**
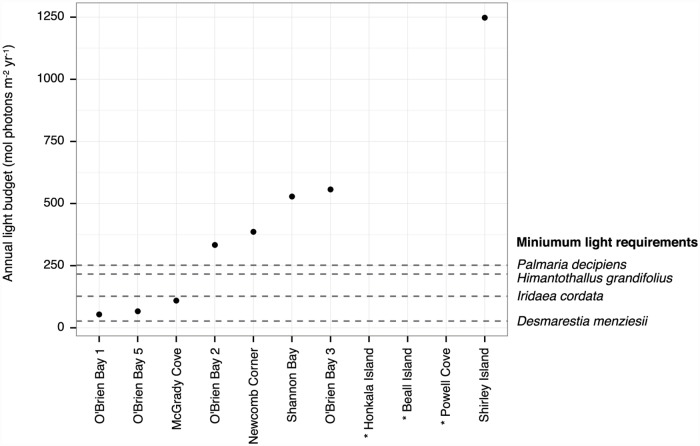
Annual Light Budgets (ALB) at the seabed. Points represent ALB recorded by light meters during the first year of deployment, and dashed lines are estimated minimum annual light requirements of four species of local algae (from Clark et al. 2013). Algae species are expected to be viable at sites where the ALB is above their minimum requirement. Light measurements are not available for three sites marked with *, but from long-term visual observation we know their annual timing of sea-ice departure and therefore approximate light budgets.
