# Supplementary material for: The Roles of Sea-Ice, Light and Sedimentation in Structuring Shallow Antarctic Benthic Communities
Source: PLoS One. 2017 Jan 11;12(1):e0168391. doi: 10.1371/journal.pone.0168391 (PMC5226713; doi:10.1371/journal.pone.0168391)
Supplement: S1 Table — The number of deployments represents the number of sediment traps deployed at one site, with 2–5 sediment traps deployed at any one time. Traps were deployed for various time intervals between 08/12/2002 and 30/12/2006. (DOCX) [file pone.0168391.s001.docx]

**S1 Table.** Sampling details of sediment traps, and minimum, mean and maximum sediment flux for each site during the study period. The number of deployments represents the number of sediment traps deployed at one site, with 2-5 sediment traps deployed at any one time. Traps were deployed for various time intervals between 08/12/2002 and 30/12/2006.

| Site | Position | Latitude (S) | Longitude (E) | Depth (m) | Number of deployments | Sediment flux (g m^-2^ d^-1^) | | |
| --- | --- | --- | --- | --- | --- | --- | --- | --- |
|  |  |  |  |  |  | Minimum | Mean | Maximum |
| Brown Bay | Inner | 66°16.823' | 110°32.483' | 6 | 33 | 0.50 | 2.53 | 10.99 |
|  | Outer | 66°16.818' | 110°32.697' | 12 | 23 | 0.14 | 1.94 | 5.30 |
| McGrady Cove | Inner | 66°16.624' | 110°34.475' | 11 | 17 | 0.16 | 0.80 | 2.64 |
|  | Outer | 66°16.522' | 110°34.279' | 11 | 15 | 0.23 | 0.88 | 2.55 |
| O’Brien Bay 1 | Inner | 66°18.732' | 110°30.887' | 9 | 30 | 0.04 | 1.57 | 6.91 |
|  | Outer | 66°18.685' | 110°30.945' | 12 | 25 | 0.05 | 0.52 | 1.78 |
| O’Brien Bay 5 | Inner | 66°18.718' | 110°33.325' | 10.5 | 20 | 0.03 | 1.05 | 5.38 |
|  | Outer | 66°18.620' | 110°33.145' | 10.5 | 18 | 0.05 | 0.58 | 2.12 |
| Sparkes Bay 1 | Inner | 66°20.970' | 110°31.720' | 7 | 10 | 1.24 | 4.58 | 8.21 |
|  | Outer | 66°21.005' | 110°31.934' | 11 | 7 | 2.28 | 4.40 | 8.84 |
| Sparkes Bay 2 | Inner | 66°20.736' | 110°32.726' | 8 | 5 | 1.22 | 2.24 | 6.07 |
|  | Outer | 66°20.834' | 110°32.644' | 10 | 8 | 0.06 | 1.53 | 3.95 |
