# Supplementary material for: The Roles of Sea-Ice, Light and Sedimentation in Structuring Shallow Antarctic Benthic Communities
Source: PLoS One. 2017 Jan 11;12(1):e0168391. doi: 10.1371/journal.pone.0168391 (PMC5226713; doi:10.1371/journal.pone.0168391)
Supplement: S2 Table — At each site we collected 16 boulders, 8 from 6 m depth and 8 from 12 m. (DOCX) [file pone.0168391.s002.docx]

**S2 Table.** Details of sampling sites and times for the surveys of biota. At each site we collected 16 boulders, 8 from 6 m depth and 8 from 12 m.

| Site | Latitude (S) | Longitude (E) | Sampling dates |
| --- | --- | --- | --- |
| O’Brien Bay 1 | 66° 18.72’ | 110° 30.88’ | 11/01/2006, 2/12/2006 |
| O’Brien Bay 5 | 66° 18.70’ | 110° 33.33’ | 12/12/2006 |
| McGrady Cove | 66° 16.62’ | 110° 34.43’ | 10/11/2006 |
| O’Brien Bay 2 | 66° 17.64’ | 110° 32.18’ | 23/11/2006 |
| Newcomb Corner | 66° 16.16’ | 110° 34.19’ | 3/02/2006 |
| Shannon Bay | 66° 16.71’ | 110° 31.28’ | 13/11/2006 |
| O’Brien Bay 3 | 66° 17.63’ | 110° 31.22’ | 9/12/2006 |
| Honkala Island | 66° 13.85’ | 110° 36.25’ | 1/12/2006 |
| Beall Island | 66° 18.12’ | 110° 27.38’ | 18/02/2006 |
| Powell Cove | 66° 15.18’ | 110° 31.83’ | 15/12/2006 |
| Shirley Island | 66° 16.77’ | 110° 28.85’ | 13/12/2006 |
