# Supplementary material for: The Roles of Sea-Ice, Light and Sedimentation in Structuring Shallow Antarctic Benthic Communities
Source: PLoS One. 2017 Jan 11;12(1):e0168391. doi: 10.1371/journal.pone.0168391 (PMC5226713; doi:10.1371/journal.pone.0168391)
Supplement: S3 Table — (DOCX) [file pone.0168391.s003.docx]

**S3 Table.** Taxa recorded in boulder survey.

| Phylum | Class | Taxon |
| --- | --- | --- |
|  |  |  |
| Bryozoa | Gymnolaemata | Arachnopsia decipiens |
|  |  | Beania erecta |
|  |  | Celleporella antarctica |
|  |  | Celleporella dictyota  Disporella sp. |
|  |  | Ellisina antarctica |
|  |  | Escharoides tridens |
|  |  | Exochella avicularis |
|  |  | Fenestrulina exigua |
|  |  | Fenestrulina crystallina |
|  |  | Fenestrulina fritilla |
|  |  | Filaguria spatula |
|  |  | Harpecia spinosissima  Idmidronea sp. |
|  |  | Inversiula nutrix |
|  |  | Lacerna eatoni |
|  |  | Lageneschara lyrulata |
|  |  | Micropora brevissima |
|  |  | Smittoidea melleata |
|  |  | Smittina rogickae |
|  |  | Toretocheilum absidatum |
|  |  | Cheilostomate bryozoan 1 |
|  |  | Ctenostomate bryozoan 1 |
|  | Stenolaemata | Tubulipora sp. |
|  |  |  |
| Cnidaria | Hydrozoa | Hydroid |
|  |  |  |
| Porifera | Demospongiae | 12 unidentified spp. |
|  |  |  |
| Chordata | Ascidiacea | Semi-colonial ascidian |
|  |  | Ascidian |
|  |  |  |
| Annelida | Polychaeta | Spirorbis nordenskjoldi |
|  |  | Upright spirorbid |
|  |  | Unidentified serpulid |
|  |  |  |
| Rhodophyta | Florideophycidae | Iridaea cordata* |
|  |  | Palmaria decipiens* |
|  |  | Gymnogongrus antarctica* |
|  |  | Plocamium cartilagineum* |
|  |  | Red encrusting algae |
|  |  | Pink coralline algae |
|  |  |  |
| Heterokontophyta | Phaeophyceae | Himantothallus grandifolius** |
|  |  | Desmarestia menziesii** |
|  |  | Brown encrusting algae |
|  |  |  |
| Chlorophyta | Ulvophyceae | Monostroma horiotii |
|  |  |  |

Notes: (a) Due to the coarse taxonomic resolution of new recruits, taxa marked with * or ** were collectively recorded as “red foliose algae” or “brown foliose algae”, respectively. (b) All Gymnolaemate bryozoa were of the Class Cheilostomate, except for Ctenostomate bryozoan 1 (Class Ctenostomate).
